# Retaining Adolescent and Young Adult Participants in Research During a Pandemic: Best Practices From Two Large-Scale Developmental Neuroimaging Studies (NCANDA and ABCD)

**DOI:** 10.3389/fnbeh.2020.597902

**Published:** 2021-01-18

**Authors:** Kate B. Nooner, Tammy Chung, Sarah W. Feldstein Ewing, Ty Brumback, Zjanya Arwood, Susan F. Tapert, Sandra A. Brown, Linda Cottler

**Affiliations:** ^1^Department of Psychology, The University of North Carolina at Wilmington, Wilmington, NC, United States; ^2^Department of Psychiatry, Rutgers, The State University of New Jersey, New Brunswick, NJ, United States; ^3^Department of Psychology, University of Rhode Island, Kingston, RI, United States; ^4^Department of Psychology, Northern Kentucky University, Highland Heights, KY, United States; ^5^Department of Psychiatry, University of California, San Diego, La Jolla, CA, United States; ^6^Department of Epidemiology, University of Florida, Gainesville, FL, United States

**Keywords:** adolescent, young adult, longitudinal, neuroimaging, retention, pandemic, developmental

## Abstract

The novel coronavirus pandemic that emerged in late 2019 (COVID-19) has created challenges not previously experienced in human research. This paper discusses two large-scale NIH-funded multi-site longitudinal studies of adolescents and young adults – the National Consortium on Alcohol and Neurodevelopment in Adolescence (NCANDA) and the Adolescent Brain Cognitive Development (ABCD) Study – and valuable approaches to learn about adaptive processes for conducting developmentally sensitive research with neuroimaging and neurocognitive testing across consortia during a global pandemic. We focus on challenges experienced during the pandemic and modifications that may guide other projects, such as implementing adapted protocols that protect the safety of participants and research staff, and addressing assessment challenges through the use of strategies such as remote and mobile assessments. Given the pandemic’s disproportionate impacts on participants typically underrepresented in research, we describe efforts to retain these individuals. The pandemic provides an opportunity to develop adaptive processes that can facilitate future studies’ ability to mobilize effectively and rapidly.

## Introduction

The novel coronavirus that hit the world in the latter part of 2019 (COVID-19) has changed the global research landscape. In the United States, more than 80% of on-site research activities stopped at some point since the pandemic began (Wigginton et al., 2020). COVID-19 has mandated changes to research protocols, particularly to large, multi-site studies that examine neurodevelopment with carefully planned timelines that require time-sensitive in-person contact and retention over many years. Here we focus on two National Institutes of Health (NIH) funded studies of adolescent and young adult development: (1) the National Consortium on Alcohol and Neurodevelopment in Adolescence (NCANDA), comprised of 831 youth, recruited at ages 12-21 years and followed across five sites in the United States, to determine the effects of alcohol use on the developing brain (Brown et al., 2015); and (2) the Adolescent Brain Cognitive Development (ABCD) Study, comprised of 11,880 children, ages 9-10 years at baseline from twenty-one sites in the United States, as they mature through adolescence (Auchter et al., 2018). The emergence of COVID-19 presented an opportunity for further collaboration between these consortia, and across disciplines, to adapt and implement developmentally sensitive protocols that ensure the safety of participants and staff, and advance knowledge of the effects of a pandemic on youth and young adult development (Holmes et al., 2020).

NCANDA and ABCD joined forces to offer suggestions on best practices for adapting assessment protocols and steps taken to retain adolescent and young adult research participants during and after the pandemic, including underrepresented minority (URM) (e.g., Black/African American, Hispanic/Latinx) individuals. To do this, we convened expert workgroups to review and make recommendations—including discussions with administrative units, principal investigators and research staff across consortia—for adhering to safety regulations while maintaining as much data collection as is feasible during the pandemic, particularly aspects that require in-person assessment [e.g., neuroimaging (Auchter et al., 2018)].

### NCANDA and ABCD Background

**FIGURE 1:**
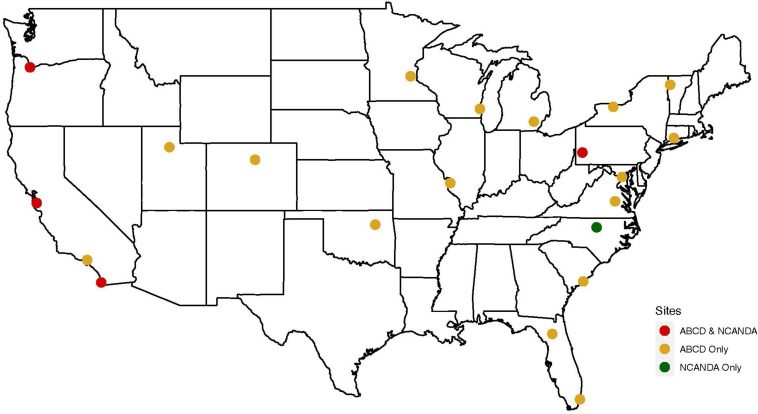
NCANDA and ABCD sites (*N* = 22 total). All located in the United States. Yellow = ABCD sites only (*n* = 17); Green = NCANDA site only (*n* = 1), Red = NCANDA and ABCD sites (*n* = 4; *note – staff and participants are separate).*

NCANDA recruited pre-adolescent to young adult participants (*N* = 831, ages 12–21 at baseline), along with their parent, in an accelerated longitudinal design at five sites across the United States (Figure 1; Brown et al., 2015). Youth complete baseline and annual in-person follow-ups to age 22, after which they are assessed by phone annually, with in-person visits at ages 24 and 27; throughout the study, mid-year phone interviews are conducted, and self-report data are collected by a project app (mNCANDA) installed on the smartphone of consenting participants (Cummins et al., in press). The NCANDA battery includes neuroimaging, neurocognitive testing, biospecimen collection (e.g., saliva, urine), and a comprehensive interview and questionnaire assessment which covers substance use, mental and physical health, education and employment, and adverse childhood experiences. For a detailed description of NCANDA, see Brown et al. (2015). The NCANDA goal is to maintain 85% or higher consortium-wide retention of those eligible at each follow-up through year 12. Among NCANDA participants, there are not cumulative differences by race/ethnicity in the completion of interview or brain scans through 5-year follow-up. More than 90% of participants across race/ethnic groups have completed 3 + assessments, which is the minimum required to statistically compute developmental trajectories.

In ABCD, participants (ages 9–10 at baseline) were recruited with a participating parent, to complete annual phenotypic assessments (Auchter et al., 2018) and biannual neuroimaging at 21 sites in the United States (Figure 1). The ABCD battery covers substance use, neurocognition, mental and physical health, culture and environment, collects biospecimens (e.g., blood, saliva, urine, hair sample, baby teeth), and uses novel technologies (e.g., Fitbit) to provide objective information on physical health, sleep, and activity level. Between in-person visits, a brief virtual mid-year assessment maintains participants’ connection with the study, updates contact information, and collects interim data on mental health and substance use. For a detailed description of ABCD, see Auchter et al. (2018). The ABCD consortium goal is to maintain 95% or higher retention at each follow-up. Since ABCD recruitment occurred in 2016–2018, the first wave of data collection has been completed, but the second wave of scans was suspended in mid-March 2020 due to pandemic-related mandated stay-at-home orders. Therefore, statistics on retention trajectories are not yet available (Auchter et al., 2018).

NCANDA and ABCD studies differ in duration, with NCANDA’s recruitment occurring in 2012–2013 and ABCD’s in 2016–2018. NCANDA and ABCD studies diverge in the number and location of sites. Four of the five NCANDA sites are also ABCD sites; however, the participants and staff do not overlap. Common features of NCANDA and ABCD include consortium-wide development of safety protocols for staff and participants to minimize infection risk, and allowance for sites to determine when any on-site assessment activity is safe. Together, the projects’ catchment areas cover 35% of the US population and illustrate broadly generalizable features that can be applied to a diverse range of projects (Brown et al., 2015; Auchter et al., 2018).

### Retention of Underrepresented Minority Participants

Though all participants may face hurdles to completing biomedical studies, due to histories of negative experiences in health care and biomedical research, and the resource-demand of participating in research studies (e.g., taking time off from work to attend sessions; navigating transportation, childcare), individuals from URM populations may face even greater difficulties participating in research studies, particularly during challenging times, such as the pandemic (Cottler et al., 1996; Striley et al., 2008; Singh et al., 2017; Sander et al., 2018). Broad representation is essential, however, to the validity, generalizability and reproducibility of research findings, and reducing bias associated with non-random dropout (Robinson et al., 2007; Poulton et al., 2015; Western et al., 2016). As ongoing longitudinal studies, NCANDA and ABCD are attuned to barriers to retention. To date, in NCANDA, there has been no evidence of differential attrition by race/ethnicity. Attrition data for ABCD are not yet available. Specific methods used by the consortia to enhance retention of URM individuals (Table 1) start at recruitment, and include, for example, leveraging community relationships to build trust, anticipating individual needs (e.g., arranging transportation), and offering flexible scheduling (e.g., multiple visits to accommodate busy schedules) (Feldstein Ewing et al., 2018; Hoffman et al., 2018; Webb et al., 2019; Kim et al., 2020).

**TABLE 1:** Challenges and Solutions for Pandemic Retention from NCANDA and ABCD.

**Challenge**	**Solution**
*Maintaining Contact with Participants and Families*	• Conduct telephone sessions between yearly visits. • Update contact information at each visit. • Use IRB approved and developmentally tailored methods to contact participants (phone, email, physical mailing, social media, birthday cards, newsletters). • Use an project-specific app to keep participants engaged and keep contact information up to date. • Contact participants using different methods, times and staff, following IRB guidelines.
*Maintaining Trust of Participants and Families*	• Explain thoroughly and regularly confidentiality and its limits. • Anticipate participant needs (e.g., offer transportation meals, childcare, reimburse travel costs). • Build rapport using Motivational Interviewing techniques to foster a positive experience, demonstrating care and respect for participants and family members. • Recognize participants’ personal contributions to the study (e.g., participation certificates) and highlight benefits of participation (e.g., via a newsletter). • Leverage community relationships to build trust and engagement, especially among underrepresented minority groups.
*Addressing Staff Needs and Concerns*	• Develop and maintain standards for troubleshooting challenges with participants, and addressing questions and concerns of participants and parents (i.e., Standard Operating Procedures adapted to COVID-19 impacts). • Prepare staff to address concerns regarding participant fears of in-person visit and possible resistance to complying with safety requirements (e.g., wearing mask, COVID-19 symptom screening 2 weeks prior to and day of in-person assessment). • Hold weekly staff meetings for research assistants to address staff burnout directly and address any staff need or concern (e.g., how to handle an observation of possible abuse or neglect, suicidality via video conference). • Provide staff with computers and internet access to work from home. • Ensure staff can access computers that are secure outside of institutional settings. • Modify assessment methods for straightforward home administration, including neuropsychological measures whenever possible. • Use video technology to bring staff together.
*Neuroimaging Session Retention*	• Use mock scan sessions to reduce pre-scan anxiety. • Obtain post-scan feedback at each visit to better understand how to improve in-scan experiences. • Accommodate flexibly a participant’s schedule.
*Pandemic-Specific Adaptations to the Protocol*	**SAFETY** • Re-arrange research rooms to ensure participants and staff are at least 6 feet apart at all times. • Add safety structures to in-person research settings (e.g., plexiglass shields separating research staff and participants; conduct in-person on-site assessments in separate rooms equipped with quality video-conferencing, so that participants and staff can remove masks during the assessment). • Explain to participants (and parents, as appropriate) how the equipment is cleaned before each use to minimize concerns. • Ensure adequate personal protective equipment (PPE) and cleaning supplies at all times. • Normalize PPE used by staff and participants in developmentally appropriate ways. For example, develop scripts that might be included in Standard Operating Procedures for research staff to use when normalizing PPE with participants; increase participant comfort with PPE by sending photos of staff with PPE prior to the visits. • Make masks available for participant use, giving masks away to participants. • Use positive body language to facilitate participant retention when PPE is worn. **PRIVACY and CONFIDENTIALITY** • Adapt study protocols to allow for survey/interview based data to be collected in private settings (e.g., online, by phone) while staff and participants are at home. • Ensure privacy during data collection by offering more flexible scheduling (e.g., evenings, weekends) and offer suggestions to participants on where to complete interviews. **ASSESSMENT MODIFICATIONS** • Add IRB approved measures to assess COVID-19 symptoms, testing, vaccine beliefs, impacts on quality of life, psychosocial changes, and healthcare use. • Prioritize the measures to be administered when participants’ time on devices is limited. • Revise protocol, with IRB approval, to limit in-person time as conditions change. • Ask adolescents (and parents, as appropriate) about experiences during research visits to continually improve procedures. • Provide participants with technology (e.g., tablets pre-loaded with protocol materials) and data plans to facilitate remote completion of assessments.
	**RETENTION** • Communicate with participants (and parents, as appropriate) regarding the safety of research sites (e.g., Centers for Disease Control [CDC]-based safety protocols) to allay fears regarding COVID-19 infection risk, and to develop accurate participant expectations regarding research participation, such as CDC-based symptom monitoring of participants and study staff. • Remain in contact with participants and families throughout pandemic to pro-actively address concerns regarding continuing participation (e.g., IRB approved methods, such as phone, e-mail, social media, study website, newsletter). • Increase flexibility in timing of visits to allow for cleaning, distancing and maximal accommodation of participant’s schedules.
*Possible Adaptations Going Forward*	• Assess transportation needs as return to in-person assessment becomes possible. • Consider providing tablets to children and families with limited internet access and/or devices to support virtual engagement/participation. • Re-structure in-person settings to achieve safe distances as conditions change (e.g., larger spaces, use of multiple rooms, use of additional PPE). • Revise cleaning protocols for in-person study areas. • Revise protocol to address participant concerns upon return to in-person settings and for ongoing remote assessment. • Revise staff-structure to address concerns based on safety/social distancing needs etc.

## COVID-19 Broad Impacts on Health and Longitudinal Research With Youth

The distribution of COVID-19 cases in the US varies markedly across states and over time, and has ranged from 386 to 4846 diagnosed cases per 100,000 persons across NCANDA and ABCD sites (Center for Disease Control and Prevention, 2020b). COVID-19 may impact participants and their families, for example, through educational disruption, changes in employment (e.g., job loss, salary changes), interrupted communication services (e.g., phone, internet), geographic relocation, change in healthcare access, and food and housing insecurity (Brock and Laifer, 2020; Brooks et al., 2020; Ghosh et al., 2020). Social/physical distancing and associated isolation might have long-term impacts on participants and their families (e.g., increased domestic violence and stress (Bradley et al., 2020); reduced contact with supports).

Pandemics such as COVID-19 have disproportionately affected URM groups (Dorn et al., 2020; Ferdinand and Nasser, 2020; Millett et al., 2020). For example, some URM individuals have experienced greater adverse COVID-19-related health, financial, and family stress impacts ranging from decreased access to medical care to greater economic impacts associated with job loss (Maggs, 2020), family members becoming ill, and navigating greater childcare needs (e.g., due to transition to “virtual” schooling). Underserved and URM groups in particular, also may be more vulnerable to COVID-19-related stress associated with social distancing and prolonged isolation (Millett et al., 2020). In addition, multi-systemic impacts (e.g., at community, interpersonal levels) may increase the chronicity of existing stressors, particularly among underserved groups in the United States (Coughlin et al., 2020; Millett et al., 2020).

The pandemic could impact health outcomes of interest in NCANDA and ABCD, such as substance use, physical and mental health, and brain and cognitive development and functioning. The protocols now include enhanced assessment of impacts on stress and emotion associated with social distancing. These are receiving additional attention in both consortia with pandemic-related supplements.

### Substance Use

NCANDA and ABCD will leverage on-going longitudinal data collection to prospectively examine the effect of pandemic-related closures of schools, public businesses and entertainment venues, and social/physical distancing practices on substance use (Dumas et al., 2020; Maggs, 2020). Of note, a study of 14–18 year-olds in Ontario, Canada during stay-at-home orders found that youth who reported substance use had increased alcohol and cannabis use relative to the pre-stay-at home period, with stress/coping motives cited most often as the reason for use (Dumas et al., 2020; Maggs, 2020). Further, certain contexts of increased adolescent alcohol use during stay-at-home periods are particularly concerning, such as solitary alcohol use, and alcohol use with parents (Dumas et al., 2020; Maggs, 2020). Importantly, these motives and contexts for substance use predict later substance-related problems, which can persist into adulthood, (Brown et al., 2008) making them important for longitudinal studies to monitor.

### Physical and Mental Health

Children and adolescents who had the COVID-19 virus or who were in contact with someone with COVID-19 are at risk for multisystem inflammatory syndrome in children (MIS-C) (European Centre for Disease Prevention and Control, 2020). Similar to COVID-19, risk for MIS-C is higher among those with lower socioeconomic status, which may disproportionally impact URM youth (Riphagen et al., 2020). The coronavirus also may have primary or secondary effects on brain structure and functioning among those infected (Ghosh et al., 2020; Mahammedi et al., 2020), which NCANDA and ABCD studies are well-positioned to monitor with their multi-modal batteries. Effects of COVID-related trauma and chronic stress may impact brain structure and functioning, particularly during critical periods of adolescent development (Zucker and Brown, 2019). Acute impacts of the pandemic, for example, in the form of stress on emotion processing and regulation could influence an individual’s performance on neuropsychological and other study measures (e.g., activity level, sleep). These potential COVID-19-related impacts highlight the importance of on-going assessment of physical and mental health conditions in NCANDA and ABCD (Sheth et al., 2017).

### Unexpected Opportunities

The pandemic has provided opportunities to gain greater insight into specific aspects of quality of life, such as healthcare access; and to enhance rapport with participants during mandated stay-at-home. Specifically, both projects added assessment of COVID-19 symptoms, testing, attitudes toward vaccination, and healthcare access. Most of these assessments were deployed within weeks of the pandemic and some will continue to be administered. Mandated stay-at-home offered the projects greater availability to some participants, who had previously been challenging to reach. Staying-at-home also provided opportunities for research staff to solidify positive connections with participants, and for participants to reinvest in their highly valued role in the project. Research compensation also held increased value for some due to the loss of other income sources.

## Adaptations to the Research Protocol in Response to the Pandemic

Adaptations to the NCANDA and ABCD research protocols (Table 1) took into account the developmental level of participants (e.g., role of the parent versus autonomous young adult), and evolved as conditions changed (e.g., stay-at-home orders lapsed or resumed). During the initial suspension of all in-person research activity (e.g., neuroimaging, biosamples), study protocols were rapidly adapted to remote versions, with Institutional Review Board (IRB) approval, while researchers and participants were at home. The remote or “virtual” assessment protocol, conducted with supervision by research staff (e.g., video chat) as needed, required new standard operating procedures for consenting and assenting, ensuring secure data transmission, and managing participant privacy within the home/remote setting. To keep participants and families informed of COVID-19-related project changes, study websites were updated and local resource information was provided.

### Confidentiality and Privacy

To maintain a high level of research integrity, maintaining confidentiality and privacy was a chief concern for study staff and participants. Each consortia developed adapted remote protocols that were HIPAA-compliant to maximize confidentiality of data collection. Maintaining rapport with participants was critical as some were in crowded quarters, necessitating creativity to complete assessments and flexibility in scheduling that afford more privacy (e.g., evenings). Providing the same high level of confidentiality and privacy helped maintain participant comfort with study participation, despite modified procedures.

### Electronic Device and Internet Access

Electronic access was a consideration for study staff and participants, as some may not have had access to devices or internet for completing assessments. For staff, laptop computers were rapidly updated to allow for secure use outside of institutional settings, and efforts were made to provide service for staff without internet service. Unreliable internet connections represented an ongoing challenge for staff, and participants and their families. For participants without video capabilities on their devices, some assessments were completed by phone. To address this challenge, a triaged approach allowed for critical assessments to be prioritized in case a participant’s time on devices was limited (e.g., shared family device). Some youth had school-provided tablets or laptops. Youth from families with fewer economic resources and from less-resourced school districts were less likely to have access to these devices and the stable internet connections needed to power them, which also disproportionally impact URM communities (Ejiogu et al., 2011). Therefore, careful consideration was taken to ensure that all participants were individually assessed to determine their needs to maximize follow-up. Sites used evolving multi-modal approaches to keep participants, especially those with limited resources, engaged. Distribution of tablets and mobile hotspots may be helpful going forward, budgets permitting.

### Maintaining Staff Morale

Staff morale and rapport with participants were key concerns, as they are cornerstones of retention. To address morale of research assistants, who are responsible for much of the day-to-day scheduling and assessments of participants and parents, consortia held weekly staff meetings to specifically address questions about protocol adaptions as well as staff burnout (Foster, 2020; Restauri and Sheridan, 2020). These weekly meetings were essential for building cohesion while working from home, understanding how staff and participants were responding to the pandemic, and modeling self-care by discussing what burnout looks like, how to best address it, and ways to find support from the consortium and other professional and personal sources (Cénat et al., 2020).

## Special Considerations for In-Person Visits During the Pandemic

NCANDA and ABCD consortia continue to carefully consider when participants can safely complete in-person research visits at each site. Timelines for in-person research appointments vary across sites and are subject to change based on the virus incidence rate which differs by region, local, state and federal regulations, and institutional requirements. While the consortium establishes minimal safety guidelines based on CDC recommendations (Center for Disease Control and Prevention, 2020a), sites have developed protocols to meet the physical distancing, sanitization and use of PPE applications. Research rooms have been re-arranged to ensure that participants and study staff are at least 6 feet apart at all times, have appropriate ventilation, and stocks of cleaning supplies and masks.

### Returning to In-Person Visits

Participants were informed in advance of the logistical changes for in person assessments. Those due for an in-person visit during pandemic-related lab closures completed remote (i.e., phone/computer) assessments within the required assessment window. At the next annual interview (i.e., if/when labs opened), sites performed neuroimaging scans, collect neuropsychological data, biospecimens, self-reports and the full phenotypic interview. The majority of the assessment battery was completed remotely, limiting in-person contact to only parts of the study that could not be done remotely (e.g., neuroimaging, biospecimens). This hybrid (remote and in-person) protocol allowed consortia to acquire approximately the same number of total scans while limiting in-person contact and maintaining well-spaced scan data for most participants. Although in-person neuroimaging stopped at all sites in March 2020, it resumed with enhanced protocols to meet COVID-19 safety guidelines in August 2020. Some sites paused or decreased in-person neuroimaging in November 2020 due to seasonal increases in rates of COVID-19.

To further address gaps in data collection between the extended follow-up intervals starting at age 24, NCANDA developed the mNCANDA app, which began collecting weekly reports of substance use and other health behaviors (e.g., exercise) in Year 6 (Cummins et al., in press). The mNCANDA app leverages this age group’s high ownership of mobile phones and willingness to engage in quick (median time: 180 sec) incentivized surveys (Cummins et al., in press). Because most youth, including URM youth, have a mobile phone even if their access to other devices (e.g., laptops) is more limited, the mNCANDA app is of particular use during the pandemic (Marler, 2018; Steinberg et al., 2018).

### Neuropsychological Testing and Biospecimen Collection

As described above, during in-person assessment, larger tables are used to maintain distance during testing sessions, and plexiglass dividers minimize direct airflow between participant and research staff. Some sites have separate testing rooms for the participant and research staff, who communicate by video-conferencing. If possible, some in-person testing (e.g., grooved peg board) may be delayed until a subsequent visit. Similarly, with biospecimen collection, additional PPE (e.g., face shields) in addition to masks, are worn by study staff to ensure greater protection for participants and study staff. Saliva and urine samples can be left in designated areas or containers by participants, so that staff can maintain physical distancing. Given the extensive use of cleaning supplies to disinfect rooms and equipment, and PPE by study staff and participants, projects need to budget for increased costs.

### Impact of PPE on Research

Both consortia and individual sites have discussed how having staff and participants wear PPE may impact retention and performance on study measures (e.g., stress associated with wearing a mask). Adolescents are at a developmental stage when concerns about their appearance and how they are perceived by their peers is highly externally oriented (Zucker and Brown, 2019). Adolescents (versus children or adults), have a greater sensitivity to peer evaluation and are highly influenced by perception of peer appraisals. Therefore, youth may be resistant to wearing PPE themselves and may be hesitant to enter a research setting where staff are wearing PPE because this could increase uncomfortable thoughts and feelings about the pandemic.

Staff wearing PPE could impact participant performance on neurocognitive and clinical assessments as well as phenotypic measures of coping and stress. As such, study staff will need to take steps to normalize PPE use both by study staff and participants to prepare participants for their visit and openly discuss at the start of each visit. Steps to normalize PPE include conducting in-person assessment in separate rooms with use of video-conference, if available, to allow communication between participant and research staff without masks. When masks are used, staff can acknowledge directly, thoughtfully, and appropriately, concerns that adolescent participants and their parents may have about wearing PPE and returning to the research setting during a pandemic. NCANDA and ABCD Coordinating Centers have provided guidance in developing basic scripts for communicating in a developmentally appropriate manner with participants, which can be tailored according to institutional guidance and IRB regulations.

## Conclusion

NCANDA and ABCD consortia have leveraged the interdisciplinary strengths of psychologists, physicians, neuroimagers, technicians, and essential research staff to implement protocol adaptations and retention strategies to safely conduct in-person research assessments with participants during a pandemic. The approaches reviewed represent evolving best practice guidelines based on two large scale longitudinal neuroimaging studies of youth and young adults that are designed to capture change across a range of domains (e.g., brain, cognition, behavior) during a critical period of development. COVID-19 required adaptions to the research protocol and has led to the collection of unique new data during this developmental period, such as attitudes toward vaccination, the emotional and stress-related impacts of prolonged social isolation, and examination of differential impacts of a pandemic across URM subgroups. In keeping with the tenets of the open science model, the goal of the NCANDA and ABCD consortia is to foster an environment of open collaboration that will enhance trust in the research process for our participants and families.

## Data Availability Statement

The datasets presented in this study can be found in online repositories. The names of the repository/repositories and accession number(s) can be found below: NCANDA.org.

## Ethics Statement

The studies involving human participants were reviewed and approved by each of the site for NCANDA and ABCD. Written informed consent to participate in this study was provided by the participants’ legal guardian/next of kin.

## Author Contributions

KN was the primary author. TC, SF, and TB contributed with writing, editing, and figures. ZA contributed with formatting, tables, and references. ST, SB, and LC contributed with overall conceptualization and substantial editing. All authors contributed to the article and approved the submitted version.

## Conflict of Interest

The authors declare that the research was conducted in the absence of any commercial or financial relationships that could be construed as a potential conflict of interest.
